# Association between weight gain during adjuvant chemotherapy for early‐stage breast cancer and survival outcomes

**DOI:** 10.1002/cam4.1207

**Published:** 2017-10-10

**Authors:** Gustavo Schvartsman, Angelica M. Gutierrez‐Barrera, Juhee Song, Naoto T. Ueno, Susan K. Peterson, Banu Arun

**Affiliations:** ^1^ Division of Cancer Medicine The University of Texas MD Anderson Cancer Center Houston Texas; ^2^ Department of Breast Medical Oncology The University of Texas MD Anderson Cancer Center Houston Texas; ^3^ Department of Biostatistics The University of Texas MD Anderson Cancer Center Houston Texas; ^4^ MD Anderson Cancer Center Houston Texas

**Keywords:** Adjuvant chemotherapy, body mass index, breast cancer, obesity, survival outcomes, weight gain

## Abstract

Obese and overweight women have an increased risk of breast cancer and worse outcomes at the time of diagnosis. Women tend to gain weight after breast cancer diagnosis and during chemotherapy for early‐stage disease, which may in turn increase risk for worse outcomes. We examined if weight gained during adjuvant chemotherapy was associated with worse survival outcomes. We queried our database for data on patients who received adjuvant third‐generation chemotherapy for early‐stage breast cancer. Univariate and multivariate analyses by Cox regression were performed for survival outcomes across three categories according to BMI variation from start to end of chemotherapy: >0.5 kg/m^2^ loss or gain and stable BMI (±0.5 kg/m^2^). We included 1998 patients in this study. Women over 50 years old and postmenopausal were more likely to lose weight during adjuvant chemotherapy, whereas women under 30 years old gained more weight (*P* < 0.001). At 1 year postchemotherapy, patients tended to return to their original weight (*ρ *= −0.3, *P *<* *0.001). On multivariate analysis, BMI increase of >0.5 kg/m^2^ compared to maintaining BMI was marginally associated with increased locoregional recurrence risk (HR: 2.53; 95% CI, 1.18–5.45; *P *=* *0.017), adjusting for grade, stage, and radiation delivery. Weight variation during adjuvant chemotherapy for early‐stage breast cancer may occur as both weight gain and weight loss in a balanced manner. Furthermore, this variation seems to be transient in nature and does not appear to significantly influence recurrence rates and overall survival.

## Introduction

A world‐epidemic, particularly in the United States 1, 2, 3, obesity has been linked with an increased risk for development of breast cancer, and possibly worse outcomes when compared to nonobese patients at the time of diagnosis 4, 5, 6, 7, 8, 9, 10.

Moreover, it has been suggested that women tend to gain weight after breast cancer diagnosis, as a result of decreased energy expenditure, depression, hormonal imbalance, and changes in body composition 11, 12, 13, 14, 15. As demonstrated previously, increase in weight within the first year after breast cancer diagnosis may potentially lead to worse outcomes, although data are conflicting 16, 17, 18, 19, 20. Weight variation can be further exacerbated when patients undergo chemotherapy for early‐stage breast cancer 20, 21. It has been suggested that overweight and obese patients who undergo neoadjuvant chemotherapy may be less likely to achieve a pathologic complete response (pCR) 22. Additionally, obesity may interfere with drug metabolism, contributing to lower response rates 23, 24.

Despite some efforts to address this matter, how weight change during adjuvant chemotherapy, when observed, may affect long‐term survival outcomes remains widely unclear, particularly regarding the possible transient nature in which this weight variation may occur. The purpose of our study was to examine how patients’ weight variations that occur during adjuvant chemotherapy for early‐stage breast cancer behave 1 year after the last infusion and how weight gain during treatment influences long‐term survival outcomes.

## Materials and Methods

Under a protocol approved by the Institutional Review Board of The University of Texas MD Anderson Cancer Center (UTMDACC, Houston, TX), we queried the UTMDACC prospectively collected Breast Cancer Management System Database for patients that met the following inclusion criteria: early‐stage (I–III) breast cancer diagnosis that received a third‐generation adjuvant chemotherapy (anthracycline and/or taxane‐based) regimen.

### Data collection

Data were extracted for patient demographics (race, age, and menopausal status), treatment data (chemotherapy and endocrine treatment, surgery type and surgical margin, and adjuvant radiation delivery), and tumor characteristics (clinical stage, estrogen receptor [ER], and progesterone receptor [PR], combined as hormone receptor status, histologic grade, and HER‐2 status). Smoking status was not available for the majority of patients and was not included. Follow‐up information for patients in the Breast Cancer Management System database is obtained every 2 years by direct review of the medical records and linkage to the MDACC Tumor Registry, which mails annual follow‐up letters to each patient registered at MDACC known to be alive to determine the patient's clinical status. Recurrence data were obtained and broken down to locoregional, distant, and contralateral recurrence. The MDACC Tumor Registry checks the Social Security Death Index and the Texas Bureau of Vital Statistics for the status of patients who do not respond to the letters.

Body mass index (BMI) was calculated by dividing weight (kg) by the square of the height (m^2^) 25, assessed by nursing staff during follow‐up visits. BMI was calculated at diagnosis, first chemotherapy date, last chemotherapy date, and 1 year after last chemotherapy date. Patients were grouped into obese (BMI ≥ 30 kg/m^2^), overweight (BMI between 25 and 30 kg/m^2^), and normal/underweight (BMI < 25 kg/m^2^). Change in BMI was computed by subtracting prechemotherapy value from postchemotherapy, and postchemotherapy value from 1 year postchemotherapy. Patients’ BMI variation from start to end of adjuvant chemotherapy was separated in three groups: (1), >0.5 kg/m^2^ loss; (2), >0.5 kg/m^2^ gain; and (3), stable BMI (maintained ± 0.5 kg/m^2^).

### Statistical analysis

Patient baseline characteristics were compared between three BMI variations during chemotherapy (three groups) utilizing Kruskal‐Wallis test for continuous variables and Chi‐square test or Fisher's exact test for categorical variables.

Five survival outcome variables since surgery, including progression‐free survival (PFS), distant recurrence‐free survival (DMFS), locoregional recurrence‐free survival (LRFS), contralateral breast cancer‐free survival (CLBCFS), and overall survival (OS), were obtained. Those without an event were censored at the time of last follow‐up. Univariate Cox regression analyses were performed. Factors with univariate *P*‐value of less than 0.15 were initially included in a multivariable model, then further reduced by backward elimination (*α* < 0.1). The same analysis was performed in the subgroup of postmenopausal women. The Bonferroni correction model was applied to account for multiple testing, in which the type I error rate of 0.05 was equally split between the five endpoints (i.e., 0.01).

## Results

We identified 2356 patients that met our study criteria, treated between 2004 and 2015. Due to unreliable weight/height abstraction, 358 patients were excluded, resulting in 1998 total patients included in this analysis. The median follow‐up time was 7.1 years (95% CI, 6.9–7.2).

The majority of patients had ductal carcinoma histology and were hormone receptor‐positive and HER‐2‐negative. Similar number of patients either lost or gained more than 0.5 kg/m^2^, or maintained their BMI. Women over 50 years old and postmenopausal were more likely to lose weight during adjuvant chemotherapy, whereas women under 30 years old gained more weight (*P *<* *0.001). At 1 year postchemotherapy, patients tended to return to their original weight (correlation between BMI change during chemotherapy and BMI change from the end of chemo to 1 year postchemotherapy: *ρ *= −0.3, *P *<* *0.001). Notably, patients that gained more than 0.5 kg/m^2^ during chemotherapy tended to lose more than half of the weight during the year postchemotherapy (*ρ *= −0.58; *P *<* *0.001). Patients that gained more than 0.5 kg/m^2^ had their weight increased by an average of 4.12 kg from first to last chemotherapy date (SD = 3.01) and lost 0.51 kg from the first day of chemotherapy to 1 year past the last chemotherapy date (SD = 5.15). Full baseline characteristics are displayed in Table 1.

**Table 1:** Baseline characteristics according to BMI change group

Variable	BMI Change (chemo start to end)			*P*‐value
	>0.5 kg/m^2^ loss [,−0.5)	Maintain ± 0.5 kg/m^2^[−0.5, 0.5]	>0.5 gain kg/m^2^(0.5,]	
	*N *=* *622	*N *=* *658	*N *=* *718	
Age at diagnosis				
<30	9 (1.4%)	8 (1.2%)	18 (2.5%)	<0.001
30–40	46 (7.4%)	82 (12.5%)	142 (19.8%)	
40–50	167 (26.8%)	227 (34.5%)	273 (38%)	
>50	400 (64.3%)	341 (51.8%)	285 (39.7%)	
Postmenopausal	426 (68.5%)	378 (57.5%)	342 (47.7%)	<0.001
BMI at diagnosis				
<18	2 (0.3%)	11 (1.7%)	6 (0.8%)	<0.001
18–25	160 (25.7%)	283 (43%)	276 (38.4%)	
25–30	186 (29.9%)	184 (28%)	234 (32.6%)	
30–35	132 (21.2%)	106 (16.1%)	131 (18.2%)	
35–40	83 (13.3%)	51 (7.8%)	45 (6.3%)	
>40	59 (9.5%)	23 (3.5%)	26 (3.6%)	
Weight change during chemotherapy^a^(Mean, SD)	−4.4 ± 3.4	0.02 ± 0.7	4.1 ± 3.0	<0.001
Weight change post chemotherapy^b^ (Mean, SD)	1.9 ± 6.1	0.4 ± 4.0	−0.5 ± 5.1	<0.001
Grade				
I–II	280 (45.2%)	319 (48.8%)	310 (43.4%)	0.127
III	339 (54.8%)	335 (51.2%)	405 (56.6%)	
Hormone receptor status				
Positive	456 (73.3%)	511 (77.7%)	548 (76.3%)	0.178
Negative	166 (26.7%)	147 (22.3%)	170 (23.7%)	
Lymph node status				
Positive	196 (31.5%)	186 (28.3%)	277 (38.6%)	<0.001
Negative	426 (68.5%)	471 (71.7%)	441 (61.4%)	
Histology				
Ductal	500 (80.4%)	519 (78.9%)	604 (84.1%)	0.078
Lobular	63 (10.1%)	69 (10.5%)	49 (6.8%)	
Other	59 (9.5%)	70 (10.6%)	65 (9.1%)	
Her‐2 status				
Positive	58 (9.3%)	91 (13.8%)	130 (18.1%)	<0.001
Negative	564 (90.7%)	567 (86.2%)	588 (81.9%)	
BRCA				
Positive	20 (14.7%)	22 (13.3%)	26 (12.4%)	0.824
Negative	116 (85.3%)	143 (86.7%)	184 (87.6%)	

Hormone receptor includes estrogen and progesterone receptor. BMI, body mass index.

a Change from first chemotherapy date to last chemotherapy date.

b Change from first chemotherapy date to 1 year after the last date.

### BMI and survival outcomes

On univariate analysis, BMI variation did not affect survival outcomes, except for worse LRFS in the group with BMI increase of more than 0.5 kg/m^2^ (Table 2). On multivariate analysis, more than 0.5 kg/m^2^ increase in BMI was only found to be associated with increased risk for locoregional recurrence (HR, 2.53; 95% CI, 1.18–5.45; *P *=* *0.017), adjusting for grade, stage, and radiation delivery (Table 3). This finding was marginally significant after reducing the alpha to 0.01, as an adjustment to multiple testing bias. To further elucidate the impact of weight gain on LRFS, we divided the “0.5 kg/m^2^ gain” category into “0.5–2.0 kg/m^2^” gain” and “>2 kg/m^2^ gain”. Interestingly, we found that LRFS was only marginally worse in the “0.5–2 kg/m^2^” group (HR, 2.684; 95% CI, 1.219–5.909; *P = *0.0142), but not for greater than 2 kg/m^2^. In absolute numbers, however, the clinical impact was low (5‐year LRFS: 99% vs. 97% in the >0.5 kg/m2 gain vs. <0.5 kg/m^2^ gain), with only 46 events among 1998 patients. Median time to locoregional recurrence was not reached yet.

**Table 2:** Univariate analysis of body mass index change during adjuvant chemotherapy and survival outcomes

	HR	95% CI		*P*‐value	5‐YS
*PFS*					
BMI change during chemo				0.347	
>0.5 loss kg/m^2^	1.06	0.72	1.57	0.752	94%
Maintain ± 0.5 kg/m^2^	1.00				94%
>0.5 gain kg/m^2^	1.28	0.90	1.84	0.171	93%
*LRFS*					
BMI change during chemo				0.024	
>0.5 kg/m^2^ loss	1.33	0.55	3.22	0.523	98%
Maintain ± 0.5 kg/m^2^	1.00				99%
>0.5 gain kg/m^2^	2.59	1.21	5.56	0.014	97%
*OS*					
BMI change during chemo				0.384	
>0.5 loss kg/m^2^	1.29	0.81	2.03	0.278	95%
Maintain ± 0.5 kg/m^2^	1.00				97%
>0.5 gain kg/m^2^	1.34	0.87	2.08	0.186	95%
*DMFS*					
BMI change during chemo				0.698	
>0.5 loss kg/m^2^	1.06	0.70	1.60		95%
Maintain ± 0.5 kg/m^2^	1.00				95%
>0.5 gain kg/m^2^	1.18	0.80	1.73		94%
*CLBCFS*					
BMI change during chemo				0.133	
>0.5 loss kg/m^2^	0.21	0.05	0.96	0.045	100%
Maintain ± 0.5 kg/m^2^	1.00				98%
>0.5 gain kg/m^2^	0.74	0.29	1.86	0.518	99%

BMI, body mass index; PFS, progression‐free survival; DMFS, distant metastasis‐free survival; LRFS, locoregional recurrence‐free survival; CLBCFS, contra lateral breast cancer‐free survival; OS, overall survival; 5‐YS, 5‐year survival HR, hazard ratio; CI, confidence interval.

**Table 3:** Univariate and multivariate analysis of locoregional recurrence‐free survival

	Univariate analysis				Multivariate analysis^a^			
	HR	95% CI		P‐value	HR	95% CI		P‐value
BMI at diagnosis				0.184				
<18	2.40	0.32	18.28	0.398				
18–25	1.00							
25–30	0.95	0.43	2.10	0.902				
30–35	1.13	0.48	2.70	0.778				
35–40	2.71	1.17	6.27	0.020				
>40	0.99	0.22	4.35	0.987				
Age				0.549				
<30	1.56	0.21	11.61	0.662				
30–40	1.61	0.74	3.49	0.232				
40–50	0.90	0.45	1.79	0.761				
>50	1.00							
Grade				0.017				0.010
I	0.00			0.986	0.00			0.986
II	0.36	0.18	0.73	0.004	0.34	0.17	0.68	0.002
III	1.00				1.00			
Hormone status (ER/PR)								
Positive	0.62	0.34	1.16	0.127				
Negative	1.00							
Her‐2 status								
Positive	1.95	0.99	3.86	0.055				
Negative	1.00							
Stage				0.060				0.023
1	1.00				1.00			
2	2.70	1.185	6.14	0.018	2.97	1.30	6.77	0.010
3	2.44	0.884	6.73	0.085	3.55	1.24	10.16	0.018
Tumor size (in cm)				0.231				
0–2	1.00							
2.1–5	1.67	0.91	3.08	0.100				
>5	1.79	0.53	6.08	0.352				
Adjuvant radiation								
Yes	0.55	0.31	0.99	0.047	0.53	0.28	0.99	0.045
No	1.00							
Type of surgery								
Mastectomy	0.74	0.29	1.88	0.532				
Lumpectomy	1.00							
Surgical margin								
Positive	0.66	0.09	4.79	0.680				
Negative	1.00							

BMI, body mass index; ER, estrogen receptor; PR, progesterone receptor; LRFS, locoregional recurrence‐free survival; HR, hazard ratio; CI, confidence interval.

a Factors with univariate *P *< 0.15 were initially included in a multivariable model then further reduced by backward elimination (α < 0.1).

**Figure 1:**
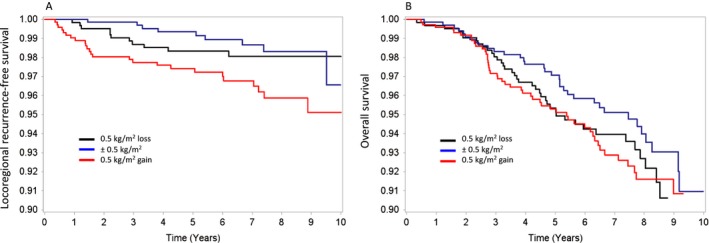
Kaplan‐Meier survival curves divided by BMI variation over time: (A) Locoregional recurrence‐free survival; (B) Overall survival.

Factors associated with worse OS were age <30 years old (HR, 2.56; 95% CI, 1.02–6.39; *P *=* *0.045), tumor stages 2 and 3, comparing to 1 (HR, 1.69; 95% CI, 1.04–2.73; *P* = 0.034 and HR, 4.65; 95% CI, 2.79–7.76; *P *<* *0.001), and tumor grade III (vs. I–II; HR, 1.99; 95% CI, 1.29–3.09; *P *=* *0.002; Table 4). Five‐year OS was similar between groups (96% overall, Fig. 1).

**Table 4:** Univariate and multivariate analysis of risk factors for overall survival

	Univariate analysis				Multivariate analysis^a^			
	HR	95% CI		*P*‐value	HR	95% CI		*P*‐value
BMI at diagnosis				0.353				
<18	1.44	0.35	5.92	0.616				
18–25	1.00							
25–30	0.77	0.48	1.22	0.267				
30–35	0.91	0.54	1.53	0.728				
35–40	1.45	0.82	2.57	0.197				
>40	1.36	0.67	2.78	0.398				
Age				0.011				0.035
<30	2.91	1.17	7.23	0.021	2.56	1.02	6.40	0.044
30–40	1.41	0.874	2.27	0.160	1.22	0.76	1.98	0.407
40–50	0.74	0.480	1.13	0.162	0.72	0.47	1.12	0.136
>50	1.00				1.00			
Grade								
I–II	1.00				1.00			
III	2.23	1.51	3.30	<0.001	1.95	1.26	3.03	0.003
Hormone status (ER/PR)								
Positive	0.53	0.37	0.77	0.001	0.62	0.41	0.94	0.024
Negative	1.00				1.00			
Her‐2 status								
Positive	1.01	0.61	1.67	0.96				
Negative	1.00							
Stage				<0.001				<0.001
1	1.00				1.00			
2	1.49	0.92	2.40	0.101	1.69	1.04	2.73	0.033
3	3.81	2.31	6.29	<0.001	4.67	2.80	7.78	<0.001
Tumor size (in cm)				0.001				
0–2	1.00							
2.1–5	1.69	1.16	2.46	0.007				
>5	3.10	1.67	5.73	<0.001				
BMI change during chemo				0.384				
>0.5 loss	1.29	0.81	2.03	0.278				
Maintain ± 0.5	1.00							
>0.5 gain	1.34	0.87	2.08	0.186				

BMI: body mass index; ER: estrogen receptor; PR: progesterone receptor; HR: hazard ratio; CI, confidence interval.

a Factors with univariate *P *< 0.15 were initially included in a multivariable model then further reduced by backward elimination (*α* < 0.1).

### BMI and survival outcomes—postmenopausal subgroup

Among the 1146 patients that were postmenopausal, BMI increase by more than 0.5 kg/m^2^ was again only found to be associated with increased risk for locoregional recurrence on multivariate analysis (HR, 3.77, 95% CI, 1.24–11.45, *P *=* *0.019), adjusting for HER‐2 status.

## Discussion

Our study was the largest cohort to our knowledge assessing weight specifically during adjuvant chemotherapy. We demonstrated that patients could equally gain, lose or maintain their weight throughout chemotherapy treatment, and, more importantly, that the weight variation seems to be transient in nature and tended to return to the patients’ baseline values after 1 year of chemotherapy completion. We also demonstrated that an increase in BMI by more than 0.5 kg/m^2^ during adjuvant chemotherapy for early‐stage breast cancer is associated with increased risk for locoregional recurrence only, though the clinical and statistical significance were marginal.

Previous reports have shown that weight gain is a common feature after breast cancer diagnosis and during chemotherapy 11, 12, 13, 16, 20, 21. The interchangeable nature of how chemotherapy and weight variation may affect one another, however, is unclear. Obesity may have a negative impact on chemotherapy efficacy, as well as patients that receive chemotherapy might have their weight further increased during treatment. This reflects on the conflicting results reported on whether weight gain after breast cancer diagnosis influences survival outcomes 16, 17, 18, 19, 20. Litton et al. 22 reported a lower rate of pCR after neoadjuvant chemotherapy in obese and overweight women, advocating for lower efficacy of therapy in this population set. Kogawa et al. 26, however, found higher rates of pCR in patients that increased their weight during neoadjuvant therapy. Kroenke et al. 16 demonstrated elevated risk of breast cancer death for nonsmoking patients who gained 0.5 kg/m^2^ or more after ≥12 months from diagnosis during follow‐up, compared with women who maintained their weight. Similarly, Camoriano et al. 20 found worse overall survival for women gaining more versus less than median weight after diagnosis. None of the aforementioned studies, however, assess weight gain specifically during the period of adjuvant therapy.

The findings of our study provide some insight on how chemotherapy may influence women's metabolism and prognosis. Chemotherapy can affect a patient's well‐being by releasing proinflammatory cytokines, decreasing energy expenditure, disrupting patients’ sleep, and hypothalamic‐pituitary‐adrenal axis, as well as by causing mood and emotional disorders 27, 28, 29, 30, 31. As seen in our study, this can affect a patient's BMI as both weight gain or loss during this period (Table 1). Due to the limited time of treatment in this setting, usually between 3 and 6 months, these changes can be transient. As evidence of this, we found a moderate negative correlation between BMI increase during chemotherapy and subsequent decrease in the year succeeding the last dose of chemotherapy (*ρ *= −0.58; *P *<* *0.001). As a consequence, except for marginally worse LRFS in patients gaining more than 0.5 kg/m^2^ in BMI, survival outcomes were not significantly affected by weight variation during this specific period.

In light of this, one could postulate two hypotheses: (1) if there is an increased risk in survival outcomes due to increase in BMI during adjuvant therapy, this risk may be mitigated by losing the weight during the year after treatment discontinuation; or (2) due to the transient nature of this weight behavior, the risk is not clinically significant.

Still, we observed an increased rate of locoregional recurrence with a BMI increase of more than 0.5 kg/m^2^. This risk was similar in the postmenopausal subgroup. It is unclear whether changes in body composition may lead to alterations in the breast stroma, both histologically and at the molecular level. Additionally, it is known that obesity is associated with higher systemic body oxidative stress and inflammatory biomarkers 32, 33, resulting in increased acute toxicity to radiation therapy 34, 35. How radiation's efficacy in breast cancer is affected by obesity, however, has not been studied. Nonetheless, this finding has minor clinical significance, particularly after splitting the group into two categories of weight gain (0.5–2.0 and >2.0 kg/m^2^) and correcting for multiple testing bias, though this could be justified by the overall low absolute number of events in this cohort.

Our study had several limitations. Its retrospective nature, albeit involving a large cohort of patients, warrants caution in data interpretation. We had incomplete data of patients’ smoking status, which has been shown to potentially influence outcomes when accounted for 16. Additionally, there is no clear guideline for appropriate cutoff selection of BMI change in this context, although 0.5 kg/m^2^ has been reported previously 16. Also, by excluding patients that received neoadjuvant therapy, in order to standardize the sequence of treatments, we may have excluded patients with more aggressive biology that required upfront systemic therapy, although the rate of triple‐negative breast cancer patients in our cohort was appropriate. The unusually low number of events overall in this study may have contributed to the absence of more significant findings, as well as the previously reported association between obesity at diagnosis and worse outcomes that were not reproduced in our study.

In conclusion, our study demonstrated that weight variation during adjuvant chemotherapy for early‐stage breast cancer may occur as both weight gain and weight loss in a balanced manner. Furthermore, this variation seems to be transient in nature and does not appear to significantly influence recurrence rates and overall survival.

## Conflicts of Interest

The authors have no relevant conflicts of interest to disclose.
